# Variants of *ST8SIA1* Are Associated with Risk of Developing Multiple Sclerosis

**DOI:** 10.1371/journal.pone.0002653

**Published:** 2008-07-09

**Authors:** Seema Husain, Cagri Yildirim-Toruner, Justin P. Rubio, Judith Field, The Southern MS Genetics Consortium, Marvin Schwalb, Stuart Cook, Marcella Devoto, Emilia Vitale

**Affiliations:** 1 Institute of Genomic Medicine and Department of Pediatrics, UMDNJ-New Jersey Medical School, Newark, New Jersey, United States of America; 2 The Howard Florey Institute, University of Melbourne, Parkville, Victoria, Australia; 3 Department of Neuroscience UMDNJ-New Jersey Medical School, Newark, New Jersey, United States of America; 4 The Children's Hospital of Philadelphia, and CCEB, University of Pennsylvania School of Medicine, Philadelphia, Pennsylvania, United States of America; 5 Department of Experimental Medicine, University La Sapienza, Rome, Italy; 6 CNR Institute of Cybernetics, Naples, Italy; Leiden University Medical Center, Netherlands

## Abstract

Multiple sclerosis (MS) is an inflammatory demyelinating disease of the central nervous system of unknown etiology with both genetic and environmental factors playing a role in susceptibility. To date, the *HLA DR15/DQ6* haplotype within the major histocompatibility complex on chromosome 6p, is the strongest genetic risk factor associated with MS susceptibility. Additional alleles of IL7 and IL2 have been identified as risk factors for MS with small effect. Here we present two independent studies supporting an allelic association of MS with polymorphisms in the *ST8SIA1* gene, located on chromosome 12p12 and encoding ST8 alpha-N-acetyl-neuraminide alpha-2,8-sialyltransferase 1. The initial association was made in a single three-generation family where a single-nucleotide polymorphism (SNP) rs4762896, was segregating together with *HLA DR15/DQ6* in MS patients. A study of 274 family trios ( affected child and both unaffected parents) from Australia validated the association of *ST8SIA1* in individuals with MS, showing transmission disequilibrium of the paternal alleles for three additional SNPs, namely rs704219, rs2041906, and rs1558793, with p = 0.001, p = 0.01 and p = 0.01 respectively. These findings implicate *ST8SIA1* as a possible novel susceptibility gene for MS.

## Introduction

Multiple sclerosis (MS) is the most common chronic inflammatory disease of the central nervous system in young adults [1], [2]. A primary feature of MS is an inability of the brain to assert its inherent restorative potential to repair damage to myelin and oligodendrocytes. However, the severe irreversible clinical dysfunction is caused by a loss of axons that seems to occur even in early stages of the disease [3], [4], [5]. The disease begins in most patients with episodes of relapsing-remitting MS, and is often followed by a prolonged period of clinical remission. With time and repeated relapses, recovery is less complete and a gradual clinical progression known as “secondary progressive MS” occurs. [6]

The mechanisms of demyelination and axonal injury are heterogeneous, complex and difficult to study [5], [7]. Although the morphology of the MS lesions can provide evidence for diagnosis, the pathophysiologies of individual plaques vary, making the disease process difficult to define. The main challenge in MS remains the understanding of pathological events that lead to initiation and evolution of the disease.

**Figure 1:**
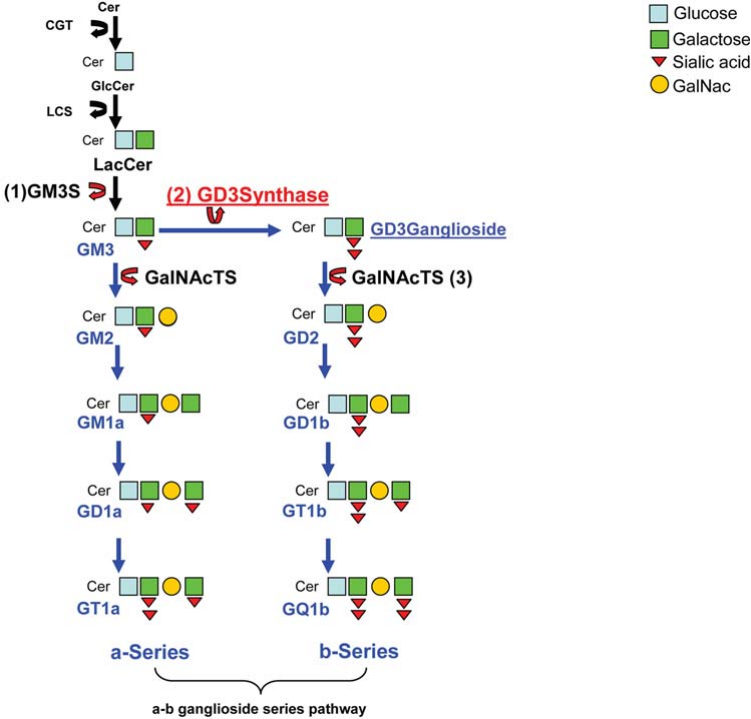
Partial ganglioside biosynthesis pathway scheme. The three key regulatory enzymes in ganglioside biosynthesis, GM3 synthase (1), GD3 synthase ((2) from the *ST8SIA1gene*), and N-acetylgalactosaminyltransferase ([3] GalNAcT) are shown. Ganglioside biosynthesis initiates with a stepwise glycosylation of ceramide (cer) to form glucosylceramide (GlcCer) and lactosylceramide (LacCer) mediated by the enzyme ceramide glucosyltransferase (CGT) and lactosylceramide synthase (LCS) respectively . The action of different sialyltransferases converts LacCer into gangliosides GM3 and then GD3G. Sequential addition of N-acetylgalactosamine (GalNac), galactose and sialic acid residues generate the a-series and b-series respectively.

While there is evidence of the involvement of environmental risk factors, epidemiological twin and adoption studies also support a genetic component in the etiology of the disease [8], [9], [10] in which multiple interacting risk loci play a role [11]. Here we provide strong statistical genetic evidence of the involvement of the *ST8SIA1* gene in conferring disease risk for MS. *ST8SIA1* encodes GD3 synthase, a ubiquitously expressed type II transmembrane protein that generates GD3 ganglioside (GD3G) by catalyzing the addition of a second sialic acid residue to its immediate precursor GM3 [12], [13], [14], [15] (Figure 1). Gangliosides are amphipathic molecules composed of a ceramide lipid anchor attached to an externally oriented oligosaccharide chain of variable length and complexity. These lipids are the primary glycoconjugates on neurons and carry most of the sialic acid present in the brain. Ganglioside biosynthesis typically begins in the endoplasmic reticulum (ER) [15] and continues in the Golgi apparatus where resident glycosyltranferases complete the process [14], [15], [16]. The functional role of GD3 synthase in important brain functions and the identification of genetic variants associated with risk of MS may provide a new opportunity to decipher the mechanism of tissue injury in this disease.

## Results

In a previous genome-wide linkage scan of a unique three-generation Pennsylvania Dutch family (PD) [17], we identified an 18 cM segment on chromosome 12p12 linked to MS in association with the HLA class II variant *HLA-DR15/DQ6*.

**Figure 2:**
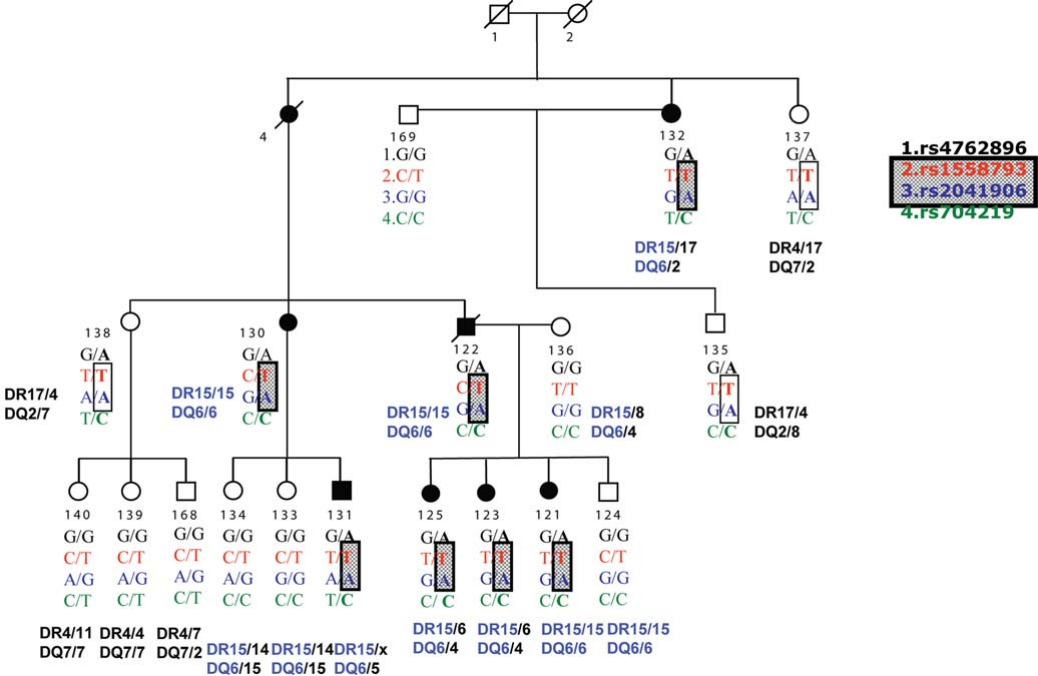
PD family pedigree showing the SNP haplotype and HLA DR15/DQ6 genotype. Closed symbols are the subjects with clinically definite multiple sclerosis (CDMS) 132, 130,122,125,123,121, and clinically possible multiple sclerosis (CPMS) 131. A detailed neurological evaluation and lab test supported the diagnosis of MS. Open symbols are the healthy subjects. The results of the SNPs analyses are reported underneath each subject. The highlighted haplotype is composed of the same SNP alleles associated to risk of MS in the Australian trios.

DNA sequence analysis of 10 (Table S1) of the 94 known genes (NCBI http://www.ensembl.org) contained in the 12p12 region linked to MS in the Pennsylvania Dutch (PD) family [17], identified a G/A polymorphism (rs4762896), at +29 in the intron 4 of the *ST8SIA1* gene. Using high throughput sequencing analyses we screened promoter, coding regions and associated intronic splice junctions of the *ST8SIA1* gene. The primer sequences and the sequence information are available upon request. We examined all seven affected as well as the 11 unaffected individuals (Figure 2). While a number of polymorphic variants were found, in the *ST8SIA1* gene we did not identify other functional polymorphisms and mutations that could have been responsible for the association and only rs4762896, clearly proved to segregate with the affected individuals in this family. The seven patients also had the *HLA DR15/DQ6* haplotype as previously described. In contrast, but in agreement with previous linkage analysis [17], three (134, 133, and 124) out of the nine at-risk unaffected individuals (Figure 2) carried the *HLA DR15/DQ6* allele but did not carry the A allele of rs4762896, while three (137, 138, and 134) with the chromosome 12 haplotype, also had the A variant but not *HLA DR15/DQ6*. The remaining three (140, 139 and 168) had neither *HLA DR15/DQ6* nor the A variant.

Given the supporting genetic data, we considered *ST8SIA1* a promising candidate risk gene for MS and initiated an independent study for genetic association of *ST8SIA1* SNPs in a large Australian MS population. MS cases used in this study were of North European Caucasian ancestry and selected based on the availability of both parents. Trios offer an advantage over case control studies since with association tests such as the transmission disequilibrium test (TDT), results are not affected by potential bias due to population stratification. The availability of parental genotypes also allows testing of specific hypotheses such as parent of origin effects, which cannot be tested in case-control samples.

We genotyped the first 209 MS trios with the previously identified SNP located in intron 4 (rs4762896) and eight additional SNPs (rs704219, rs2041906, rs1558793, rs2193177, rs1019332, rs2160536, rs1861606, rs272882) that span the entire *ST8SIA1* gene. Based on prior evidence of differential parental transmission [18], [19] of genetic variants in MS, we decided to test transmission of our candidate genes SNPs separately in mothers and fathers to the affected children.

Given the initial sample size of 209 trios, we estimated that we had >80% power to detect an odds ratio (OR) of 1.8 or higher at p = 0.05.

Statistical analysis of data from these samples indicated significantly increased transmission of paternal alleles for rs704219 (p = 0.006), rs2041906 (p = 0.004) and rs1558793 (p = 0.03). All the other SNPs had TDT p-value >0.05 (data not shown). We then included 65 additional MS trios from Tasmania genotyped for the same three SNPs. The results showed the same trend and the combined data supported our initial observation (p = 0.001 for rs704219, p = 0.01 for rs2041906 and p = 0.01 for rs1558793 when considering only the paternal transmission). The SNP showing the strongest association with MS in the Australian trio population was rs704219 or C118T, a synonymous coding SNP in exon 5 of the *ST8SIA* gene. The result for rs704219 was due to overtransmission of the minor allele T from the unaffected fathers to offspring affected with MS (frequency of the T allele was 0.26 in transmitted, and 0.15 in un-transmitted paternal chromosomes). The odds-ratio for risk of MS based on the observed frequency of the T allele in transmitted and un-transmitted paternal chromosomes was 2.02 (95% CI 1.28–3.18).

HLA DR15/DQ6 typing was available for 273 children in the Australian trios. We tested interaction between the three SNPs typed in the extended Australian cohorts (rs704219, rs2041906, and rs1558793) and HLA DR15/DQ6 in two different ways. First, we divided the trios into DR15 positive (145) and negative (128) and repeated the TDT analysis separately in the two groups. Results were very similar between the two groups and in agreement with those observed in the two groups combined although less significant as expected given the smaller sample size (DR15-positive patients: fathers p-values: 0.01, 0.09, 0.02, mothers p-values: 0.74, 0.23, 0.41; DR15-negative patients: fathers p-values: 0.04, 0.06, 0.02, mothers p-values: 0.64, 0.88, 0.73). In addition, we performed a case-only analysis of interaction by comparing the three SNPs allele and genotype frequencies in the two groups of patients (145 DR15 positive and 128 DR15 negative). There were no significant differences in either allele or genotyping frequencies between the two group (all p-values >0.05). Thus results in the Australian cohort do not show evidence of statistical interaction between the two loci.

Finally, haplotype analysis identified a partially shared haplotype between the PD family and the AU trios (Table 1 and figure 2), composed by the T allele of rs1558793 and the A allele of rs2041906.

**Table 1:** 

SNP	Allele	Freq-T in 209 trios	Freq-NT in 209 trios	P value in 209 trios	Allelic OR in 209 trios	Freq-T in 274 trios	Freq-NT in 274 trios	P value in 274 trios	Allelic OR in 274 trios	Freq-T (fathers only)	Freq-NT (fathers only)	*P* value (fathers only)	Allelic OR
rs704219	C	290 (0.75)	307 (0.80)	0.14	1.29	380 (0.74)	408 (0.80)	0.04	1.36	178 (0.74)	205 (0.85)	0.0022	2.02
	T	96 (0.25)	79 (0.20)			133 (0.26)	105 (0.20)			63 (0.26)	36 (0.15)		
rs2041906	G	187 (0.50)	211 (0.56)	0.08	1.29	258 (0.51)	291 (0.58)	0.04	1.30	113 (0.51)	137 (0.62)	0.02	1.56
	A	190 (0.50)	166 (0.44)			244 (0.49)	211 (0.42)			108 (0. 49)	84 (0.38)		
rs1558793	T	266 (0.72)	246 (0.67)	0.11	0.78	356 (0.72)	330 (0.67)	0.07	0.78	166 (0.77)	143 (0.66)	0.01	0.59
	C	104 (0.28)	124 (0.33)			139 (0.28)	165 (0.33)			51 (0.23)	74 (0.34)		

Freq-T: Frequency in transmitted chromosomes

Freq-NT: Frequency in non transmitted chromosomes

OR: odds-ratio

## Discussion

The results of two independent studies reported here provide evidence of a relationship between *ST8SIA1* gene variants and the development of MS. The first analysis identified a SNP, rs4762896, at +29 of *ST8SIA1* intron 4 in a three-generation family of MS individuals who also carried the *HLA DR15/DQ6* haplotype. This SNP is included in the linkage critical region of the PD family. Involvement of *ST8SIA1* was supported in a second study of 274 MS trios where paternal transmission of three additional SNPs in this gene, rs704219, rs2041906 and rs1558793, was associated with MS (p = 0.001, p = 0.01 and p = 0.01 respectively). In the AU cohort, the increased MS risk was related to preferential paternal transmission of the T allele for SNP rs704219 on 12p12. Although there are various mechanisms that can account for paternal transmission, our results suggest that *ST8SIA1* may be an MS susceptibility gene possibly regulated by genomic imprinting. In this regard a recent study using a computational method for predicting the genome-wide imprinting status of human genes was recently reported [20] and this identified an imprinting effect between two genes, RBP5 and ABCC9, on 12p13 and 12p12 respectively, the precise region of the *ST8SIA1* gene.

The variants described here are not rare mutations, rather they are polymorphisms that are common in normal populations. Our data strongly indicate that sporadic and familial MS share at least one common genetic susceptibility and that studies of familial MS will help our understanding of sporadic MS, as well as confirm the genetic heterogeneity already reported [21]. Our results show that certain *ST8SIA* variants are more common in MS patients from AU and affect disease risk when paternally transmitted with an odds-ratio of 2.02 for the minor allele (T) of rs704219. Consequently, the effect of rs704219 on disease risk could be greater than that previously reported for risk associated variation in the genes for *IL2RA* and *IL7RA* (OR = 1.2) [11], [22], [23].

In conclusion, our data support a role for the *ST8SIA1* gene as a contributing factor in MS. Our studies had the advantage of identifying *ST8SIA1* in a unique single multiplex family affected by MS. We were then able to examine SNPs of this gene in a collection of sporadic MS cases and their parents from Australia. The study confirmed a role for *ST8SIA1* variants in this MS population and indicated preferential paternal transmission. While some [24] have found a preferential transmission of MS from affected fathers to their offspring, others [25] have not confirmed these results. Parental effect has been recently reported as a feature of the MHC as shown by the maternal influence in disease etiology [26]. Our study used trios in which parents were unaffected and prior identification of a specific risk gene variant had been made.

Outside of the immunoregulatory system, this is the first gene extensively involved in neuronal function and membrane structure to be implicated with MS. Having identified a specific protein and its variants associated with MS allows a new approach towards understanding the molecular physiopathology of the disease. Further studies are needed to confirm and expand upon our results.

## Methods

### Sample collection

The blood samples were collected in preservative-free ACD tubes under an IRB-approved protocol. DNA was extracted using the QIAmp DNA extraction kit (QIAGEN Inc. USA). The detailed clinical data of this family are reported elsewhere [17]. In summary six patients had clinically definite MS (CDMS-132, 130, 122, 125, 123, 121; only one had clinically probable MS (CPMS-131) after neurological evaluation (history, neurological exam, laboratory testing). The phenotypic expression and temporal profile of the disease were quite varied with regard to age of onset (24 to 33 years), clinical course (four patients were relapsing-remitting, two were secondary progressive, one was primary progressive) and anatomical location of the lesions (cerebral [six patients], optic nerve [five], cerebellum [five], brain stem [three], and spinal cord [six]). All had abnormal magnetic resonance imaging (MRI) scans consistent with MS and four of them also had abnormal cerebrospinal fluid (CSF) profiles also consistent with MS [27].

Genomic DNAs from 209 sporadic MS trio families (affected child, unaffected parents) from the Australian State of Victoria and 65 similar MS trios from the island State of Tasmania were genotyped. All patients included in the study were diagnosed as having either clinically definite MS or laboratory-supported definite MS or definite MS according to standard clinical criteria [28], [29], [30]. Recruitment and phenotyping of the Australian MS families were conducted by members of The Southern MS Genetics Consortium; Trevor Kilpatrick, Simon Foote, Helmut Butzkueven, Bruce Taylor, Niall Tubridy, Mark Marriott, Caron Chapman, Melanie Bahlo, Terry Speed and Jim Stankovich. All subjects were recruited according to guidelines approved by either The Melbourne Health Human Research Ethics Committee or the Southern Tasmanian Human Research Ethics Committee.

### SNP analysis

We performed SNPs analyses in the family using a Beckman Coulter CEQ 8000 system. The regions of interest in the *ST8SIA1* gene were PCR amplified. Primer 3 software was used to design the primers. Genomic DNA (100ng) in 25 µl PCR reaction mixture of 1X buffer (Tris-CL, KCL, (NH4)2 SO4, 15mM Mgcl2; pH8.7), 1 X Q-solution, 200 µM of dNTP, 20pmole of each primer and 2.5U of Taq DNA polymerase (QIAGEN Inc. USA). The PCR mixture was denatured for 5 min at 94°C and cycled 31 times (94°C for 30 sec, 55°C for 1.15 min and 72°C for 15 sec), followed by a 10 min extension at 72°C. Post reaction template was cleaned up by directly adding 2 U SAP (Roche) and 1U Exo I (MBI Fermentas), to 6ul of PCR product and incubating at 37°C for 1 hour and 75°C for 15 min. The standard protocol of CEQ SNP-primer extension reaction kit was used: 100ng template DNA, 1pmole SNPs Interrogation Primers, 11 µl of SNP-primer extension premix, to a final volume of 20 µl. The reaction mixture was thermocycled at 96°C 10 sec, 50°C 5 sec and 72°C 30 sec for 25 cycles. Additional clean up of the extended products was done by incubation with 1U SAP at 37°C for 1 hour and 75°C for 15 min; 0.5 µl of the purified SNP reaction to 0.5 µl size standard 80 (P/N 608395) and 39 µl of sample loading solution, to final volume of 40 µl for each in the CEQ sample plate (P/N 609801) and loaded. We used DNAs NA07057 and NA06990 from CEPH/UTAH pedigree 1331 (Coriell Cell Repositories) as normal controls. The Australian MS trio families were genotyped using the SEQUENOM platform at The Australian Genome Research Facility

### Genes Sequences

We designed intronic primers flanking each of the exons for all the genes located in the critical interval, using Primer3 software. PCR amplification was performed using a QIAGEN kit and GC Rich system (Roche) for GC rich sequences. We examined amplified products on 1% agarose gels and purified them for sequencing using QIAquick PCR Purification Kit (QIAGEN). We sequenced PCR products using the CEQ Dye Terminator Cycle Sequencing with Quick start kit (Beckman Coulter) and analyzed them on Beckman Coulter CEQ 8000 Genetic analysis system. We sequenced both strands of each product using DNA from all the family members. Control DNAs C1 (NA07057) and C2 (NA06990) from CEPH/UTAH pedigree 1331 (Coriell Cell Repositories) was sequenced as well. Analysis was done by Chromas Sequence Analysis software.

### Statistics

Association of SNP alleles with disease status was tested by means of the transmission disequilibrium test using the software Unphased [31]. Frequencies of transmitted and nontransmitted alleles from parents to affected children were estimated from parental chromosomes and used to calculate allelic odds-ratios and their confidence intervals.

## Supporting Information

Table S1 table gene's
(0.07 MB DOC)
