# Supplementary material for: Variants of ST8SIA1 Are Associated with Risk of Developing Multiple Sclerosis
Source: PLoS One. 2008 Jul 9;3(7):e2653. doi: 10.1371/journal.pone.0002653 (PMC2440423; doi:10.1371/journal.pone.0002653)
Supplement: Table S1 — table gene's (0.07 MB DOC) [file pone.0002653.s001.doc]

| Gene  (a) | Locus  (b) | Definition  (c) | Location  (d) | Sample ID Sequenced (e) | Function  (f) | Size  (g) | General  (h) |
| --- | --- | --- | --- | --- | --- | --- | --- |
| **1. ST8SIA1** | **NM_003034** | **GD3 synthase** sialyltransferase 8 (alpha-N-acetylneuraminate: alpha-2,8-sialytransferase,) ST8SIA1 | **12 p 12.1** | **132,137,**  **138,130,**  **122,136,**  **135,140, 139, 168, 134, 133, 131, 125, 123, 121 and 124. C1 and C2** | Involved in the production of GD3 and GT3 from GM3 | 133,716 bps  356 aa  40519 Da  Exons:5 | **Gangliosides are membrane-bound glycosphingolipids containing sialic acid. Ganglioside GD3 is known to be important for cell adhesion and growth of cultured malignant cells. The protein encoded by this gene is a type II membrane protein that catalyzes the transfer of sialic acid from CMP-sialic acid to GM3 to produce gangliosides GD3 and GT3. The encoded protein may be found in the Golgi apparatus and is a member of glycosyltransferase family 29.** |
| **2. ARHGDIB** | **NM_001175** | **Rho GDP** dissociation inhibitor (GDI) beta | **12p12.3** | **121, 131 and 136. C1 and C2** | Regulates the GDP/GTP exchange reaction of the RHO proteins by inhibiting the dissociation of GDP from them, and the subsequent binding of GTP to them. | 19,613 bps  201aa  22,988 Da  Exons:6 | **Rho guanine nucleotide dissociation inhibitor (GDI) 2 during apoptosis is often accompanied by their relocalisation between cellular compartments [Rho GDI 2 ]. Data suggest that D4-GDI of Rho family GTPase may be regulated during apoptosis through the caspase-3 mediated cleavage of the GDI protein.** |
| **3.SURB7** | **NM_004264** | **SRB7** suppressor of RNA polymerase B homolog (yeast) | **12p11.23** | **121, 131 and 136. C1 and C2** | Component of an RNA polymerase II holoenzyme; gene with protein product | 7,200 bps  144 aa  15564 Da  Exons:4 | [HIV-1 protein interactions](http://www.ncbi.nlm.nih.gov/RefSeq/HIVInteractions/). HIV-1 Tat interacts with the RNA polymerase II holoenzyme, which includes SRB7, during Tat-mediated transactivation of the HIV-1 LTR. |
| **4.MRPS35** | **NM_021821** | **Mitochondrial ribosomal protein S35** | **12p11** | **121, 131 and 168. C1 and C2** | Catalyze protein synthesis within the mitochondrion | 45,500 bps  323 aa  36844 Da  Exons: 8 | **Mammalian mitochondrial ribosomal proteins are encoded by nuclear genes and help in protein synthesis within the mitochondrion. This gene encodes a 28S subunit protein.** |
| **5.SSPN** | **NM_005086** | **Sarcospan** (Kras oncogene-associated gene) | **12p11.2** | **121, 131 and 168. C1 and C2** | SSPN gene is expressed in a variety of tissues with highest levels in muscle, where alternative splice variants have been observed. The function of this gene is unknown | 37,367 bps  243 aa  26618 Da  Exons:3 | **Isoform 1 is expressed exclusively in heart and skeletal muscle. Isoform 2 is expressed exclusively in brain, heart, skeletal muscle, thymus, prostate, testis,ovary, small intestine, colon, and spleen** |
| **6.BHLHB3** | **NM_030762** | **basic helix-loop-helix** domain containing, class B, 3.**Gene Aliases** DEC2, SHARP-1,SHARP1 | **12p11.23-p12.1** | **121, 131 and 168. C1 and C2** | A corepressor complex containing CtIP/CtBP facilitates RBP-Jkappa/SHARP-mediated repression of Notch target genes | 4,888 bps  482 aa  50497 Da  Exons: 5 | **Basic helix-loop-helix (bHLH) transcription factors (e.g., DEC1, also called BHLHB2) are related to Drosophila hairy/enhancer of split proteins. They are involved in the control of proliferation and development during differentiation, particularly in neurons.** |
| **7.STK38L** | **NM_015000** | **serine/threonine kinase 38 like** . **Gene aliases:** NDR2; KIAA0965 | **12p11.23** | **121, 131 and 169. C1 and C2** | NDR1 and NDR2 serine-threonine kinases are regulated by mob proteins | 81423 bps  464 aa  54003 Da  Exons: 14 | **Involved in the regulation of structural processes in differentiating and mature neuronal cells The NDR1 and NDR2 kinases were incorporated into HIV-1 particles and were cleaved by the HIV-1 protease** |
| **8. KRAS2** | **NM_004985** | **v-Ki-ras2** Kirsten rat sarcoma 2 viral oncogene homolog | **12p12.1** | **121, 131 and 168. C1 and C2** | Ras proteins bind GDP/GTP and possess intrinsic GTPase acitivity | 45,675bps  189 amino acids  21656 Da  Exons: 6 | **This gene, a Kirsten ras oncogene homolog from the mammalian ras gene family, encodes a protein that is a member of the small GTPase superfamily. A single amino acid substitution is responsible for an activating mutation. The transforming protein that results is implicated in various malignancies, including lung adenocarcinoma, mucinous adenoma, ductal carcinoma of the pancreas and colorectal carcinoma. Alternative splicing leads to variants encoding two isoforms that differ in the C-terminal region** |
| **9. CDKN1B** | **NM_004064** | **cyclin-dependent kinase inhibitor 1B (p27, Kip1)** | **12p13.1-p12** | **121, 131 and 168. C1 and C2** | Involved in G1 arrest. May mediate TGF beta-induced G1 arrest. Binds to and inhibits complexes formed by cyclin E-CDK2, cyclin A-CDK2, and cyclin D1-CDK4. Interaction with nucleoporin NUP50 is required for nuclear import and for degradation of phosphorylated p27Kip1 after nuclear import (By similarity). | 4995 bps  198 aa  22073 Da  Exons:3 | **This gene encodes a cyclin-dependent kinase inhibitor, which shares a limited similarity with CDK inhibitor CDKN1A/p21. The encoded protein binds to and prevents the activation of cyclin E-CDK2 or cyclin D-CDK4 complexes, and thus controls the cell cycle progression at G1. The degradation of this protein, which is triggered by its CDK dependent phosphorylation and subsequent ubiquitination by SCF complexes, is required for the cellular transition from quiescence to the proliferative state** |
| 10. C1R | NM_001733 | Complement component (3b/4b) receptor 1 | 12p13 | 121, 131 and 169. C1 and C2 | **The complement components exist in blood as precursors of enzyme subunits. There are 2 principal pairs of complexes: one has C3 as substrate and is made up of 1 subunit from the classic pathway and 1 provided by the alternative pathway; the second has C5 as substrate and again has subunits provided by both pathways. C1 exists as a complex of C1q, C1r, and C1s. It is activated by complexing with antigen or an acidic macromolecule such as DNA.** | **2900 bps**  **705aa**  **83,000 Da**  **Exons: 1** | **The complement complexes have multiple subunits: one carrying the active site, one that binds to the cell surface, and one that binds to substrate. The steps in the complement cascade have similarities to those in the coagulation cascade. Furthermore, many enzymes have the same pattern of activation and multimeric structure with specific function of the several subunits. C1r is enzymatic, in the alternate pathway, factors B and D are enzymatic but deficiency states are unknown. C1r is a single-chain glycoprotein. Upon activation, it is cleaved into an A chain and a B chain which are held together by a disulfide bond. The B chain contains the catalytic portion of the enzyme.** |

**Table 1.** Genes sequenced in the MS PD family

a -gene list; b - gene locus name; c- gene name’s definition; d - chromosome location of the genes sequenced; e – samples ID of the individuals sequenced for each gene, C1 and C2 are NA07057 and NA06990 respectively from CEPH/UTAH pedigree 1331 (Coriell Cell Repositories); g – function related with the genes analyzed; h – general function associated with the genes sequenced. (All the primers to amplify these genes are in tables a-j )
